# The Effect of Environmental Conditions on the Physiological Response during a Stand-Up Paddle Surfing Session

**DOI:** 10.3390/sports6020025

**Published:** 2018-03-22

**Authors:** Yair Suari, Ben Schram, Adva Ashkenazi, Hadas Gann-Perkal, Lev Berger, Meshi Reznikov, Shmuel Shomrat, Einat Kodesh

**Affiliations:** 1 School of Marine Sciences, Ruppin Academic Center, Michmoret 40297, Israel; advaashkenazi00@gmail.com (A.A.); gphadas@gmail.com (H.G.-P.); levberger1989@gmail.com (L.B.); meshiraz88@gmail.com (M.R.); shmuel2003@gmail.com (S.S.); 2 Water Based Research Unit—Bond Institute of Health and Sport, Bond University, Gold Coast, QLD 4226, Australia; bschram@bond.edu.au; 3 Physical Therapy Department, Faculty of Social Welfare and Health Sciences, University of Haifa, Haifa 31905, Israel; ekodesh@gmail.com

**Keywords:** stand up paddle board, physiology, surfing

## Abstract

Stand Up Paddleboard (SUP) surfing entails riding breaking waves and maneuvering the board on the wave face in a similar manner to traditional surfing. Despite some scientific investigations on SUP, little is known about SUP surfing. The aim of this study was to investigate the physiological response during SUP surfing sessions and to determine how various environmental conditions can influence this response. Heart rate (HR) of an experienced male SUP surfer aged 43 was recorded for 14.9 h during ten surfing sessions and synced with on board video footage to enable the examination of the effect of different surfing modes and weather conditions on exercise intensity. Results indicated that the SUP surfer’s HR was above 70% of HR_max_ during 85% of each session, with the greatest heart rates found during falls off the board (~85% HR_max_) and while paddling back to the peak (~83% HR_max_). Total time surfing a wave was less than 5%, with the majority of time spent paddling back into position. Wind speed positively correlated with HR (*r* = 0.75, *p* < 0.05) and wave height negatively correlated with wave caching frequency (*r* = 0.73, *p* < 0.05). The results highlight the aerobic fitness for SUP surfing, where wave riding, paddling back to the peak, and falls appear to be associated with the greatest cardiovascular demand and demonstrate that environmental conditions can have an effect on the physiological response during SUP surfing sessions.

## 1. Introduction

Surfing is a popular water sport in which the surfer rides a moving wave towards the shore. There are many surfing styles, including various board types, no board at all (i.e., body surfing), and the implementation of different stances [1]. Surfing’s global popularity arose when the Hawaiian Olympic swimmer, Duke Kahanamoku, demonstrated it during the 1930’s and it has since grown to become an Olympic sport in the 2020 Olympic Games.

One style of surfing in particular which has grown exponentially in popularity is SUP. This style of surfing, in which one stands upright on a surfboard and propels it using a single paddle, became popular during the mid 2000’s and has been described as one of the fastest growing sporting activities in the world [2,3]. One of the reasons for SUP’s popularity is the fact that it is relatively easy to learn. In contrast to traditional surfing, SUP uses a bigger board and a paddle, allowing better buoyancy, stability, and more power per stroke. The oversized board facilitates rapid improvements in beginner paddlers of all age groups when compared with other surfing variants [4,5]. 

Surfing an SUP is essentially the same as traditional surfing where, during a typical session, the surfer will repeatedly paddle to the take-off area (known as the “peak”), position themselves correctly, catch a wave by powerful strokes, ride the wave while maneuvering on the wave wall, and then return back to the take-off area [6].

Despite SUP’s popularity, minimal scientific investigations have been performed on the physiological demands associated with its participation. Research has shown that novice paddlers typically paddle at around 60–80% of their maximum heart rate [4]. When compared to other upper limb dominant water-based sports (surfing, dragon boat racing, and canoeing), elite SUP athletes display a similar peak exercise oxygen consumption of ~45.5 mL kg^−1^ min^−1^ [7]. Another article examined the physiological demands of distance paddling during an SUP marathon race and found paddlers were at 80–100% of their maximal heart rate for the majority of the race and that participants who covered the least distance did not necessarily finish before those who covered a greater distance [8]. Findings from this study suggested that tactics in regard to maximizing and understanding environmental conditions play a role in the outcome of these distance races and, in a similar manner, environmental conditions are known to have an impact on exercise intensity in other water sports [9,10,11].

Despite sharing many similarities with traditional surfing, the physiological response in SUP surfing is believed to be different for several reasons. One of these reasons being that the lower profile in the water in traditional surfing may allow the surfer to be less affected by wind while paddling. As SUP surfing is performed standing up, the surfer is more exposed to wind velocity, which is expected to increase physiological demands. In addition, the technique used to return to the take off point in surfing, known as the ‘duck dive’, cannot be performed in SUP surfing due to the floatation and size of the larger boards, requiring the rider to push over the breaking waves when paddling back to the takeoff zone. In traditional surfing, it is known that wave height negatively correlates with aerobic intensity, while wave period positively correlates with it [12].

Given the differences between traditional surfing, SUP surfing, and SUP distance paddling, and the lack of scientific research in SUP surfing, the aim of this investigation was twofold. The primary aim was to quantify the physiological demand of an SUP surfing session and compare it to the physiological demands of traditional surfing. The secondary aim was to determine the impact which environmental conditions may have on the physiological response of the rider. It is hypothesized that the physiological demand of SUP may be greater than what is reported in traditional surfing due to the larger profile of the SUP surfer being more affected by the environmental conditions. 

## 2. Materials and Methods

An experienced 43 year old male amateur SUP surfer (43 years, height = 1.68 m, weight = 76.5 kg, BMI = 26.6 kg/m^2^) with 30 years of traditional surfing experience and 4.5 years of SUP surfing experience was monitored for a total of 14.9 h in a total of ten surfing sessions. Prior to the sessions being conducted, the subject performed two maximal aerobic exercise tests: the first test on a cycling ergometer (General Electric CASE, Milwakee, Wi, USA) performing HR_max_ of 181, VO_2_ Peak of 51.4 mL kg^−1^ min^−1^), and the second test on a hand ergometer (Technogym top XT pro, Cesena, Italy) one week later (HR_max_ of 180 and VO_2_ Peak of 47.5 mL kg^−1^ min^−1^). The study was approved by the institutional ethics committee (University of Haifa 812015), and written consent to participate was gained by the subject.

Each SUP surfing session was recorded using an Intova Sport HD waterproof video camera (Intova, Tukwila, WA, USA) attached to the SUP to determine the activity profile of the session. To determine the cardiovascular demands of the surfing session, a telemetry heart rate monitor (Suunto Ambit 3 sports, Vantaa, Finland) was worn by the subject. Heart rate data was divided into zones with 5% increments based on the HR_max_ from the initial maximal aerobic capacity testing. Following each session, the participant completed a questionnaire regarding the perceptions of the sea conditions and gave a subjective account of the session (data acquisition is demonstrated in the supplementary material).

Analysis of the captured video footage was conducted by visually identifying activity modes (Table 1) during each surfing session. This method of analysis has been used before in surfing to determine activity profiles [13,14]. Subtitle editing software was used to conduct the analysis [15], with the changes in activity subsequently synced with the captured HR data. In this way, a time series was constructed, showing the type of physical activity and corresponding HR at any given moment during each SUP session.

Data regarding weather and sea conditions were collected and synchronized with the time series. Wind velocity was recorded using a weather station located ~8 km north of the surfing spot where most of the surfing sessions were conducted. Wave height and period data were obtained from a Global Sea Level Observing System [16] sea level observing station operated by the Israel Oceanographic and Limnological Research Institute ~8 km north of the peak. Sea roughness was not measured; therefore, choppiness was estimated by the surfer (on a scale of 1–5), along with additional data collected in a post-session questionnaire completed within one hour of the session.

All statistical analyses were completed using the Matlab [17] statistical package. Due to the temporal correlation during surfing sessions and the fact that the data was not normally distributed, no parametric tests were used, and medians are reported for heart rate. A comparison of mean HR between surfing modes was conducted using the Kruskal Wallis test, and to aid in visualization of the results of the physiological response for each surfing mode, violin plots were created using the kernel distribution function in Matlab. Significance of the correlation between environmental and surfing parameters was conducted using the Spearman test with the alpha level set to a *p* value ≤ 0.05. 

## 3. Results

**Figure 1:**
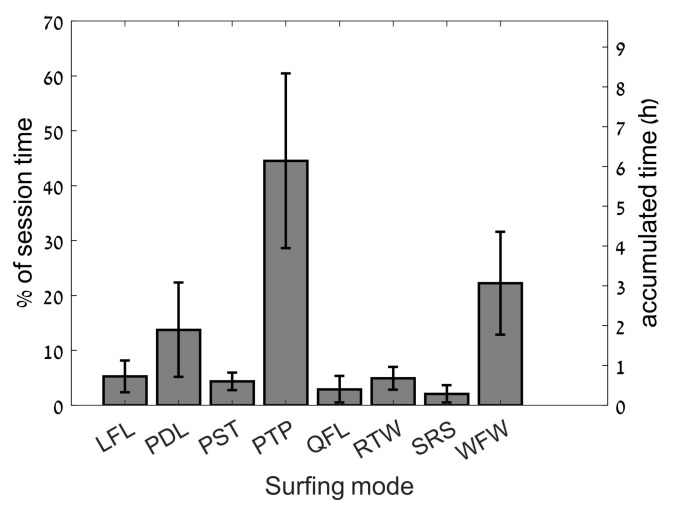
Time spent in each of the surfing modes relative to each session and overall time of all sessions. Results expressed as mean ± standard deviation.

A total of ten surfing sessions were recorded, totaling 14.9 h. The activity profile of the SUP surfing sessions is detailed in Figure 1 below. During this period, 40.51 min were spent riding waves (RTW mode), with a total of 230 waves surfed. The total time physically surfing a wave was 4.78%, while the rest of the time was spent in modes not strictly related to catching waves, including paddling and waiting for waves. 

**Figure 2:**
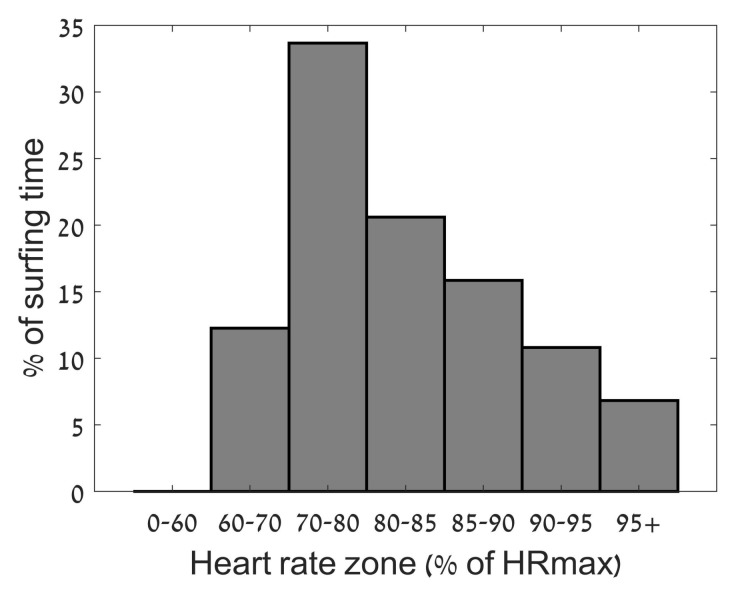
Results of the heart rate data expressed in zones relative to surfing time.

The physiological response to the session can be seen in Figure 2 below. In total, the subject’s heart rate was between 60% and 80% of HR_max_ for 53% of the recorded sessions, with the remaining 47% above 80% of HR_max_. The subject’s heart rate was recorded above 90% HR_max_ for 2.7 h (18%) of the 14.9 h.

**Figure 3:**
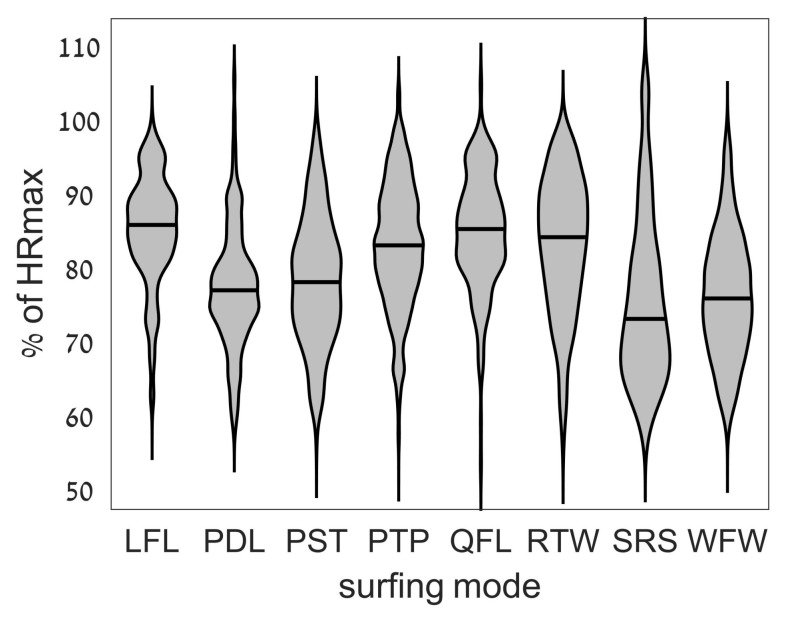
Violin plot of heart rate during each surfing mode. For each surfing mode, the frequency distribution of heart rate measurements is displayed in the form of the gray area width per % of HRmax (*Y*-axis), while the black horizontal line indicates the median. The distribution was calculated using the kernel distribution function.

Figure 3 shows the average heart rate for each individual surfing mode during the surfing sessions. Significantly greater heart rates were found during both Quick Fall and Long Fall (QFL and LFL; median of 155 and 154 bpm; 85.0% and 85.5% HR_max_) respectively, *p* < 0.01). Both riding waves (RTW, 152 bpm, 83.9% HR_max_) and Paddling to Peak (PTP, 150 bpm, 82.7% bpm) were associated with a heart rate response significantly greater than general Paddling (PDL, 139 bpm, 76.8% HR_max_), Powerful Strokes (PST, 141 bpm, 77.9% HR_max_), and Sitting Rest (SRS, 132 bpm, 72.9% HR_max_).

**Figure 4:**
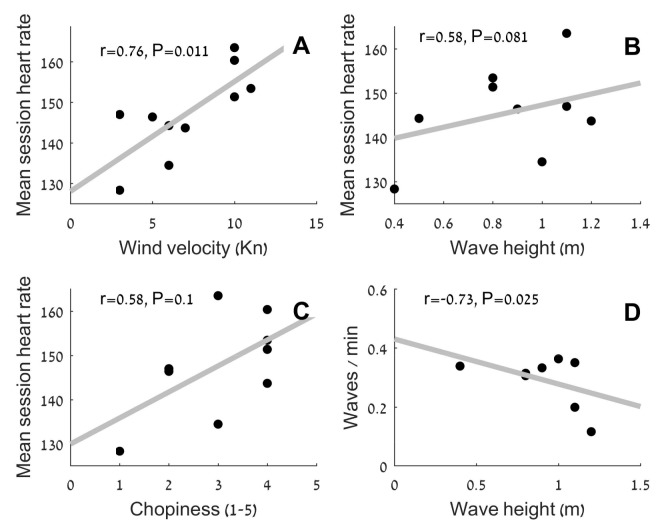
Correlation between environmental and physiological parameters. (**A**) (Top left) mean session heart rate and wind velocity; (**B**) (Top right) mean session heart rate and wave height; (**C**) (Bottom left) mean session heart rate and sea choppiness; (**D**) (Bottom right) correlation of number waves caught per minute and wave height.

During the surfing sessions, there were variable conditions with wave heights ranging from 0.4 to 1.4 m, wind velocity ranging from 3 to 13 knots with gusts up to 30 knots, and variable choppiness conditions as rated on the subjective ratings. The effect of the environmental conditions relative to cardiovascular demand can be seen in Figure 4A–C below. A significant correlation was found between mean session HR and wind velocity, while a weak correlation was found between cardiovascular demand, wave height, and the subjective rating of chopiness. A significant negative correlation was found between wave height and the number of waves caught per minute (Figure 4).

## 4. Discussion

The aim of this investigation was to profile the physiological demands of SUP surfing and determine the influence of environmental conditions. Our results indicate that during SUP surfing, the surfer heart rate is above 70% HR_max_ for ~80% of the session and positive correlations were found between wind velocity and physiological demand (HR), with the greatest heart rates elicited during riding waves and paddling while facing the waves. The results from this study suggest that SUP surfing requires high levels of aerobic fitness and also highlights the influence that some environmental conditions can have on the physiological response of the SUP surfer.

The physiological demands measured by % of HR_max_ recorded in this study appear to be greater than findings from previous research in surfing. Research has shown that heart rate will, on average, range from between 64% and 84% of HR_max_ heart rate max [6,13] in traditional surfing, similar to the 80.3 ± 9.1% of HR_max_ found in this study. The duration of traditional surfing sessions, however, ranges from 20 min competitive heats, (84% HR_max_) [6] to 2 h training sessions (66 ± 6.7% HR_max_) [14]. The longer duration of surfing in this study may highlight the fact that paddling an SUP without the ability to duck-dive may be more physiologically demanding than traditional surfing. 

In addition, 18% of the SUP surfing sessions were recorded as being above 90% of HR_max_. Despite Mendez-Villanueva and Bishop ([6], 25%) and Barlow ([12], 12.4%) finding that for the duration a traditional surfer’s heart rate exceeded 90% HR_max_, the longer duration of the SUP surfing sessions equates to a much greater physiological demand. It is thought that this may be due both to the greater influence of the environmental conditions on the standing SUP rider and the inability to duck dive, as discussed previously.

The greatest heart rates were seen during time spent in the water after falling off the board, then being tasked to regather their board and paddle and resume the standing position. This is all occurring while being hit by oncoming waves, risking hitting the ocean floor, occasionally comprised of reef or rocky outcrops. Of note is the peak heart rates which occurred while sitting resting (SRS). This appears to be a recovery strategy of the rider under high levels of fatigue and exertion in an attempt to allow the heart rate to subside. Anecdotally, the most demanding phase of SUP surfing is attempting to match the speed of oncoming waves with powerful strokes. This was not found to be physiologically demanding in this study, however, with powerful strokes (PST) recoding a median HR of 141 bpm. This may be due to the short duration of these powerful strokes with a reliance on the anaerobic system and the fact that a greater stress level is associated with the risk of hitting the sea floor and being held under the water after falling off the board. In addition, the elevation in heart rate from the quick strokes performed to catch a wave may have a short latency, which is displayed in an elevated heart rate while physically riding waves (RTW ~84% HR_max_) as it is assumed that riding a wave would not be overly challenging for the cardiovascular system. 

Environmental conditions (e.g., wave height, wave period, and perceived wave height) and their effect on surfer physiology were characterized by Barlow et al. [12] in traditional surfers. This study found a lower heart rate when wave height increased, probably due to the reduced wave catching frequency. In contrast, our study demonstrated that wave height, wind velocity, and perceived choppiness were all positively correlated with a higher heart rate (*p* < 0.05). This may be attributed to the different posture of the surfing activities. Traditional surfing is mostly performed lying down, while SUP surfing is mostly performed standing up. The standing up posture requires high stability [18,19] and higher demands of postural muscles in a dynamic, unstable environment, something which would be magnified with larger waves and more choppiness. Given the surface area of an SUP surfer who is paddling while standing, a greater influence of wind speed would also be expected. Wind velocity is known to increase with height above sea level [20], and friction with air is proportional to the square of wind velocity [21]. All these factors might provide an additional physiological demand for an SUP surfer in order to compensate for the additional effects of the wind.

Although most of the surfing session is not spent riding waves, surfers attempt to achieve as much surfing time as possible. It is often speculated that catching waves is easier on an SUP [22] and therefore, SUP surfers spend more time riding waves. This assumption sometimes causes clashes between SUP surfers and traditional surfers, who claim that SUP surfers “steal” their waves. Being an important parameter, several scholars measured the percent of session time spent riding waves. Mendez-Villanueva et al. [23] performed a time lapse analysis of professional traditional surfers during 25 min heats at a surfing competition and calculated that a mean of 3.8% of the time was spent on the wave. Farley et al. [13], sampling 20 min heats at two separate competitions and Barlow et al. [12] sampling 60 recreational surfing sessions, both showed that 8.1% of the session time was spent riding waves. Meir et al. [24] performed a similar analysis on one hour sessions of 21.1 years old recreational surfers and reported approximately 5% of the total time was spent riding waves, in agreement with the results of this study. Although this finding is probably highly dependent upon surfer ability and environmental conditions [12], it was a surprising finding that net time on the wave in SUP surfing was not significantly higher than the time reported in traditional surfing.

The single SUP surfer used in this study could be viewed as a limitation and therefore the results may not be representative of the physiological response of all SUP surfers. It is, however, a unique study which highlights the physiological demands during each mode of SUP surfing which warrants more research. Further research could expand into exploring the influence of environmental conditions such as wind velocity and choppiness and wave height on traditional surfing. The results from this study suggest that an SUP surfer could benefit from aerobic training given the high demand of the aerobic system during this activity. The role of the anaerobic system in SUP surfing is also an area for future study, with the results of this study showing that it may play an important role in the ability to catch waves, despite not being one of the most physiologically demanding aspects.

## 5. Conclusions

SUP surfing is an aerobic activity characterized by moderate to vigorous aerobic intensity with bouts above the anaerobic threshold during wave riding and while crossing the wave. SUP surfing intensity appears to be highly dependent on environmental conditions, specifically wave height and wind velocity. This dependency is thought to be amplified by the standing posture adopted compared to the prone position and lower profile in the water associated with traditional surfing.

## Figures and Tables

**Table 1:** Surfing activity modes: acronym and description.

Mode Name	Acronym	Specification
Paddle	PDL	Regular paddling, not facing incoming waves.
Powerful strokes	PST	5–10 powerful strokes prior to catching a wave.
Paddle to peak	PTP	Paddling back to the peak against incoming waves.
Wait for wave	WFW	Static standing while waiting for a wave with up to 10 strokes for maintaining the position.
Sitting rest	SRS	Static sitting during rest period.
Riding the wave	RTW	The entire ride, from the moment the wave was caught until post wave paddling begins.
Quick fall	QFL	Less than 15 s, does not include periods of being caught beneath the waves. Ends when the surfer is upright once again.
Long fall	LFL	More than 15 s, often includes periods of being caught beneath the waves. Ends when the surfer is upright once again.

## Supplementary Materials

The following are available online at http://www.mdpi.com/2075-4663/6/2/25/s1, Video S1: Synced video including surfing modes and heart rate values.

## References

[1] Booth D.G. Surfing. https://www.britannica.com/sports/surfing.

[2] Technavio Corp. (2016). Global Stand Up Paddle Board Marked 2016–2020.

[3] Schram B., Furness J. (2017). Exploring the Utilisation of Stand up Paddle Boarding in Australia. Sports.

[4] Ruess C., Kristen K.H., Eckelt M., Mally F., Litzenberger S., Sabo A. (2013). Stand up Paddle Surfing-An Aerobic Workout and Balance Training. Procedia Eng..

[5] Schram B., Hing W., Climstein M. (2016). The physiological, musculoskeletal and psychological effects of stand up paddle boarding. BMC Sports Sci. Med. Rehabil..

[6] Mendez-Villanueva A., Bishop D. (2005). Physiological aspects of surfboard riding performance. Sport. Med..

[7] Schram B., Hing W., Climstein M. (2016). Profiling the sport of stand-up paddle boarding. J. Sports Sci..

[8] Schram B.L., Hing W.A., Climstein M., Furness J.W. (2017). A Performance Analysis of a Stand-Up Paddle Board Marathon Race. J. Strength Cond. Res..

[9] Chamari K., Moussa-Chamari I., Galy O., Chaouachi M., Koubaa D., Hassen C.B., Hue O. (2003). Correlation between heart rate and performance during Olympic windsurfing competition. Eur. J. Appl. Physiol..

[10] Castagna O., Vaz Pardal C., Brisswalter J. (2007). The assessment of energy demand in the new olympic windsurf board: Neilpryde RS:X^®^. Eur. J. Appl. Physiol..

[11] Vogiatzis I., Spurway N.C., Wilson J., Boreham C. (1995). Assessment of aerobic and anaerobic demands of dinghy sailing at different wind velocities. J. Sports Med. Phys. Fit..

[12] Barlow M.J., Gresty K., Findlay M., Cooke C.B., Davidson M.A. (2014). The Effect of Wave Conditions and Surfer Ability on Performance and the Physiological Response of Recreational Surfers. J. Strength Cond. Res..

[13] Farley O.R., Harris N.K., Kilding A.E. (2012). Physiological Demands of Competitive Surfing. J. Strength Cond. Res..

[14] Secomb J.L., Sheppard J.M., Dascombe B.J. (2015). Time–Motion Analysis of a 2-Hour Surfing Training Session. Int. J. Sports Physiol. Perform..

[15] Monteiro R.B., Hansen N.M., Goyne T. (2011). Aegisub—An open source subtitle composer.

[16] Woodworth P.L., Player R. (2003). The Permanent Service for Mean Sea Level: An Update to the 21^st^Century. J. Coast. Res..

[17] MathWorks Inc. (2013). Matlab and Statistics toolbox.

[18] Elling A., Kranz J., Leikert A., Tresh T. Effects of a Four-Week Stand up Paddleboard Program on Static Balance in College Students. Proceedings of the 13th Annual Celebration of Undergraduate Research and Creative Performance.

[19] Willson J.D., Dougherty C.P., Ireland M.L., Davis I.M. (2005). Core stability and its relationship to lower extremity function and injury. J. Am. Acad. Orthop. Surg..

[20] Li Z.S., Ni J.R., Mendoza C. (2004). An analytic expression for wind-velocity profile within the saltation layer. Geomorphology.

[21] Inoue T., Okayama T., Teraoka T., Maeno S., Hirata K. (2016). Wind-tunnel experiment on aerodynamic characteristics of a runner using a moving-belt system. Cogent Eng..

[22] Krom M.D., Emeis K.-C., Van Cappellen P. (2010). Why is the Eastern Mediterranean phosphorus limited?. Prog. Oceanogr..

[23] Mendez-Villanueva A., Bishop D., Hamer P. (2006). Activity profile of world-class professional surfers during competition: a case study. J. Strength Cond. Res..

[24] Meir R.A., Lowdon B.J., Davie A.J. (1991). Heart Rates and Estimated Energy Expenditure During Recreational Surfing. Aust. J. Sci. Med. Sport.

